# *Chlamydia* Persistence: A Survival Strategy to Evade Antimicrobial Effects *in-vitro* and *in-vivo*

**DOI:** 10.3389/fmicb.2018.03101

**Published:** 2018-12-12

**Authors:** Maria Emilia Panzetta, Raphael H. Valdivia, Hector Alex Saka

**Affiliations:** ^1^CIBICI-CONICET, Departamento de Bioquímica Clínica, Facultad de Ciencias Químicas, Universidad Nacional de Córdoba, Córdoba, Argentina; ^2^Department of Molecular Genetics and Microbiology, Duke University School of Medicine, Durham, NC, United States

**Keywords:** *Chlamydia* persistence, penicillin-induced persistence, gamma interferon-induced persistence, *Chlamydia* evasion of antimicrobial stimuli, aberrant reticulate bodies, *in-vivo* implications of *Chlamydia* persistence, *Chlamydia* persistence inducers

## Abstract

The *Chlamydiaceae* comprise a group of highly adapted bacterial pathogens sharing a unique intracellular lifestyle. Three *Chlamydia* species are pathogenic to humans: *Chlamydia trachomatis, Chlamydia pneumoniae*, and *Chlamydia psittaci*. *C. trachomatis* is the leading bacterial cause of sexually-transmitted infections and infectious blindness worldwide. *Chlamydia pneumoniae* is a major cause of community-acquired atypical pneumonia. *C. psittaci* primarily affects psittacine birds and can be transmitted to humans causing psittacosis, a potentially fatal form of pneumonia. As opposed to other bacterial pathogens, the spread of clinically relevant antimicrobial resistance genes does not seem to be a major problem for the treatment of *Chlamydia* infections. However, when exposed to stressing conditions, like those arising from exposure to antimicrobial stimuli, these bacteria undergo a temporary interruption in their replication cycle and enter a viable but non-cultivable state known as persistence. When the stressing conditions are removed, *Chlamydia* resumes replication and generation of infectious particles. This review gives an overview of the different survival strategies used by *Chlamydia* to evade the deleterious effects of penicillin and IFNγ, with a focus on the different models used to study *Chlamydia* persistence, their contribution to elucidating the molecular basis of this complex phenomenon and their potential implications for studies in animal models of infection.

## Introduction

The *Chlamydiaceae* are a family of Gram-negative obligate intracellular bacteria comprising 11 species pathogenic for a variety of animals (Elwell et al., 2016), 3 of which are human pathogens: *Chlamydia trachomatis, Chlamydia pneumoniae*, and *C. psitacci*. The main impact on human health is caused by *C. trachomatis*, which leads to a variety of oculo-genital and perinatal infections. Based on the antigenic properties of the major outer membrane protein MOMP, *C. trachomatis* strains can be classified into different serovars (Stephens et al., 1987; Baehr et al., 1988; Gomes et al., 2007). Serovars A-C are the etiologic agents of trachoma, the leading cause of infectious blindness worldwide (Stocks et al., 2014). With an estimate of 131 million new cases per year, *C. trachomatis* serovars D-K are the main bacterial cause of sexually-transmitted infections (STI) globally (Newman et al., 2015). Notably, up to 70–90% of these infections may be asymptomatic and long-lasting (Stamm, 1999; Gonzales et al., 2004), leading to serious complications such as pelvic inflammatory disease, ectopic pregnancy, and infertility in women (Lan et al., 1995; Westrom, 1995; Haggerty et al., 2010). *C. trachomatis* serovars D-K also cause inclusion conjunctivitis in adults, and perinatal infections such us ophthalmia neonatorum and chlamydial pneumonia in infants (Rönnerstam et al., 1985; Schachter et al., 1986; Stenberg and Mardh, 1991; Darville, 2005; Hammerschlag, 2011). The more invasive serovars L1-L3 are the cause of a less frequent form of STI called lymphogranuloma venereum, a systemic illness characterized by inguinal lymphadenopathy and/or severe proctitis/proctocolitis (Herring and Richens, 2006; White, 2009). *C. pneumoniae* is a widespread pathogen of the human respiratory tract, causing bronchitis, community-acquired atypical pneumonia and asthma exacerbation (Grayston et al., 1993; Asner et al., 2014). *C. psitacci* is the etiologic cause of psittacosis, a zoonotic disease that can be transmitted to humans upon close contact with a variety of birds, most frequently *Psittacidae* (cockatoos, parrots, parakeets and lories) or *Columbiformes* (pigeons) and recently, exposure to equine placental material has also been found as a risk factor for transmission (Polkinghorne and Greub, 2017). Clinical symptoms of psittacosis include high fever, chills, headache, myalgia, non-productive coughing, respiratory distress and may be fatal if untreated (Beeckman and Vanrompay, 2009).

**Figure 1:**
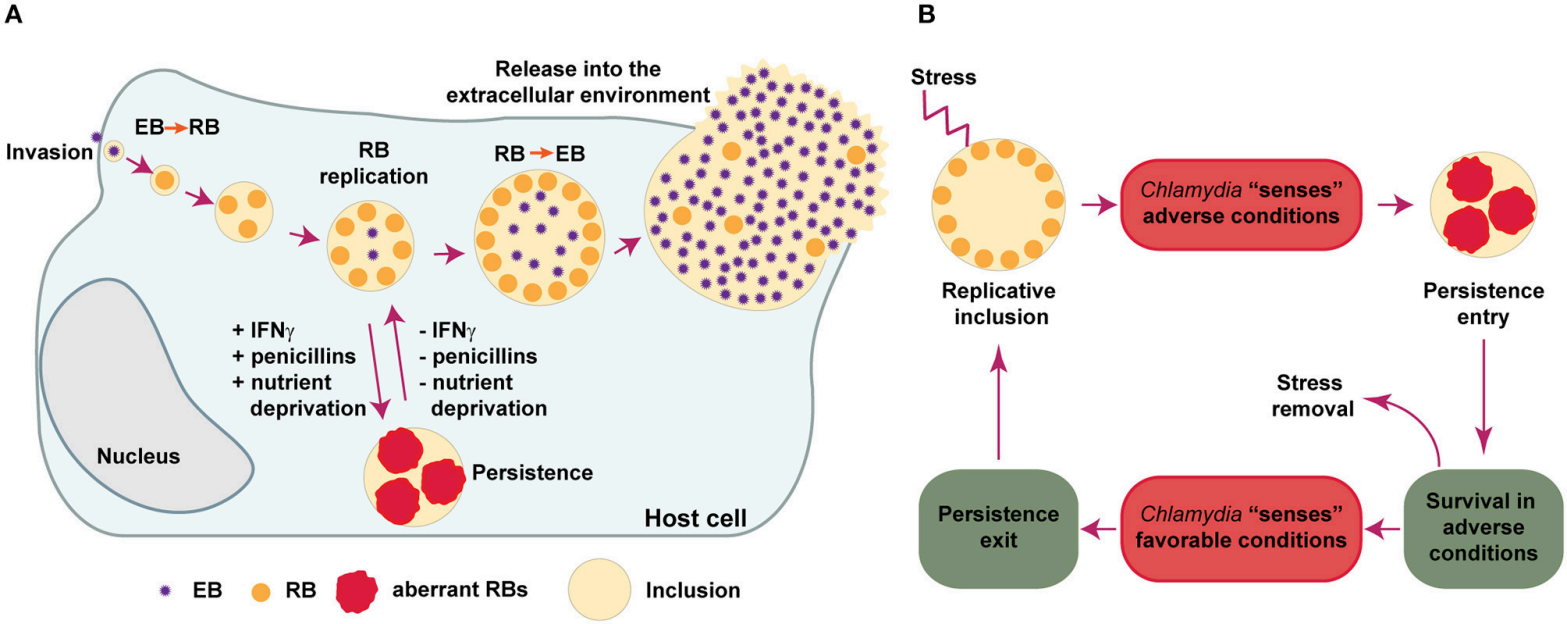
*Chlamydia* developmental cycle. **(A)** *Chlamydia* are obligate intracellular bacteria that undergo multiple developmental forms with distinct morphological and functional properties. The infectious elementary bodies (EB) are internalized in the host cells and are confined to a membrane-bound vacuole, termed an “inclusion.” Soon after invasion, EBs differentiate into replicative but non-infectious reticulate bodies (RBs), which actively divide. Around mid-cycle, RBs begin to asynchronously differentiate back into EBs and are finally released (by cell lysis or extrusion of intact inclusions) into the extracellular environment, where they can infect neighboring cells. If during replication *Chlamydia* is exposed to stressing conditions, like those caused by gamma-interferon (IFNγ), penicillins or deprivation of essential nutrients, the bacteria enter into a long-lasting, viable but non-cultivable state known as “*Chlamydia* persistence,” which is typically associated to the presence of enlarged, aberrant RBs. When conditions are again favorable, the persistence state is reversed and normal replication ensues. **(B)** This scheme summarizes different events that *Chlamydia* may use in order to successfully evade antimicrobial effects triggered during infection *in-vitro*. First, *Chlamydia* “senses” different types of stresses and then respond by entering into a temporary, reversible interruption in the replication cycle (termed “*Chlamydia* persistence” or “*Chlamydia* stress response”). In this state, *Chlamydia* adapts to adverse conditions by prioritizing cell functions required for long-lasting survival. When the bacteria sense that the stressing condition has ceased, they exit from persistence and resume normal replication and generation of infectious progeny. The *Chlamydia* factors and the molecular mechanisms required for the successful execution of each one of these steps remain poorly elucidated.

All members of the *Chlamydiaceae* are highly successful intracellular parasites that undergo a propagation cycle involving developmental forms with clearly different morphological and functional properties. The elementary bodies (EBs) are very small (ca. 0.2 μm in diameter), infectious, environmentally resistant and non-replicative. The reticulate bodies (RBs), on the other hand, are larger (ca. 0.8 μm in diameter), non-infectious, labile in the extracellular environment and actively replicative. EBs are pre-loaded with virulence factors including type III secretion effectors involved in internalization into the host cell, while RBs are enriched in proteins involved in nutrient uptake, ATP generation and translation (Saka et al., 2011). Upon attachment to epithelial cells, EBs are internalized and confined into a vacuole, termed an inclusion. At early times post-infection, EBs differentiate into RBs, which start replicating as the inclusion gradually expands. During this process, *Chlamydia* manipulates host cell pathways to acquire essential nutrients and avoid interaction with degradative organelles like lysosomes. At mid-cycle, some RBs begin to differentiate back into EBs asynchronously. At late stages post-infection the inclusion contains mostly EBs, which are finally released to the extracellular environment either by host cell lysis or extrusion of inclusions. In the extracellular environment, EBs can infect neighboring cells and continue propagating (Saka and Valdivia, 2010; Elwell et al., 2016). If during replication *Chlamydia* encounter non bacteriocidal stress conditions, like those arising from exposure to certain antibiotics, cytokines or nutrient deprivation, the bacteria respond by markedly reducing RBs division and production of infectious particles, being able to rapidly resume to normal replication once the stressing conditions are removed (Figure 1A). This response is referred to as chlamydial persistence or chlamydial stress response, as it allows these bacteria to survive for long periods of time in cell culture in presence of unfavorable growth conditions (Schoborg, 2011; Byron, 2012; Elwell et al., 2016).

As opposed to other bacterial pathogens, for which acquisition of resistance genes by lateral gene transfer confer resistance to antimicrobial drugs and represent a major concern for public health (Chatterjee et al., 2018), *Chlamydia* have so far remained susceptible to all anti-chlamydial antibiotics. An exception to this widespread susceptibility is the well-characterized tetracycline resistance island in the pig pathogen *Chlamydia suis*, which so far has not spread to *Chlamydia* species affecting humans (Seth-Smith et al., 2017). In fact, there are no reports of naturally-acquired, stable antibiotic resistance in *Chlamydia* strains recovered from human samples (Sandoz and Rockey, 2010). Interestingly, despite wide availability of effective drugs and apparent lack of antibiotic resistance mechanisms, *Chlamydia* continue to be widespread pathogens, notorious for their ability to cause long-lasting, persistent infections (Byron, 2012). Perhaps as a result of their long evolutionary history as obligate intracellular parasites, *Chlamydia* seem to have evolved unique mechanisms to resist antimicrobial effects triggered by the host innate and adaptive immune responses. In this review, we will address the current knowledge about the strategies used by these microorganisms to resist antimicrobial stimuli triggered by penicillins and IFNγ in cell culture or *in-vivo* models of infection, with a focus in *Chlamydia* persistence.

## Early Evidence of *Chlamydia* Persistence as a Response to Antimicrobial Stimuli

The view that *Chlamydia* only alternates between EB and RB developmental forms is an oversimplified view of its life cycle. Already in 1950, Weiss discovered that *Chlamydia muridarum* and *C. felis* (then known as the murine and feline pneumonitis viruses, respectively), displayed an enlarged, abnormal morphology upon exposure to the antibiotic penicillin (Weiss, 1950). Similar observations were reported for *C. trachomatis* LGV and *C. psittaci* (by then named lymphogranuloma and meningopneumonitis viruses, respectively) (Hurst et al., 1953; Tamura and Manire, 1968; Matsumoto and Manire, 1970). A seminal report from Galasso and Manire (1961), showed that penicillin was able to reduce the generation of *C. psittaci* infectious progeny in HeLa cells to values as low as 0.1% of the original titer. Surprisingly, these investigators found that normal replication rates recovered quickly upon penicillin removal, even after 3 and a half months of continuous presence of the antibiotic in cell culture. Morphological analysis by transmission electron microscopy, demonstrated that in the presence of penicillin, *C. psittaci* inclusions contained greatly enlarged RBs, while normal morphology was recovered upon removal of the antibiotic (Matsumoto and Manire, 1970). We now know that those early observations are in line with the presence of enlarged, aberrant RBs (aRBs), typically found when *Chlamydia* undergo nutrient deprivation, exposure to certain antibiotics, INFγ or other compounds/conditions that impose a stress to these microorganisms (Byron, 2012). The aRBs observed *in-vitro*, may be considered the morphological manifestation of *Chlamydia* entry into a persistent state, characterized by lack of cultivability while conditions are unfavorable, and followed by a quick return to normal replication upon removal of the stressor. In this context, the persistent state may function as a resilience pathway triggered in *Chlamydia* to deal with stressing conditions elicited by antimicrobial stimuli. Thus, it is reasonable to imagine that in order to execute such response, these bacteria should follow a sequence of events involving: (i) sensing of unfavorable conditions, (ii) entry into a persistent state, (iii) survival in adverse conditions, (iv) sensing of favorable conditions, (iv) exit from persistence, and (v) resume to normal production of infectious progeny (Figure 1B). The mechanistic and molecular basis underlying each one of the mentioned events, remain poorly understood. However, we know that under the persistent state, *Chlamydia* slows down DNA replication and continues to transcribe genes, but stops dividing, becoming viable but non-cultivable (Ouellette et al., 2006; Muramatsu et al., 2016). This is frequently accompanied by the presence of enlarged aRB forms, which retain their ability to transition into infectious EBs once conditions are again favorable. It is important to mention, however, that a functional approach to the definition of *Chlamydia* persistence as a reversible interruption of the productive cycle (Byron, 2012), is probably more appropriate than a morphological definition, since aRBs are not always observed (Schoborg, 2011).

## Diverse Stimuli Trigger *Chlamydia* Persistence *in-vitro*

A variety of stimuli induce *Chlamydia* persistence including several antibiotics, gamma-interferon (IFNγ), deprivation of essential nutrients (i.e., iron, amino acids, glucose), heat-shock, components found on cigarette smoke, exposure to adenosine, infection with chlamydiaphage, co-infection with Herpes Simplex Virus or Porcine Epidemic Diarrhea Virus (Beatty et al., 1994b; Hsia et al., 2000; Wiedeman et al., 2005; Huston et al., 2008; Pettengill et al., 2009; Vanover et al., 2010; Schoborg, 2011; Prusty et al., 2012; Schoborg and Borel, 2014). The diversity of stimuli that can elicit persistence, suggests that *Chlamydia* does not respond equally to all of them. This notion is supported by several studies showing that the transcriptional responses triggered are significantly different according to the persistence model used (Mathews et al., 2001; Belland et al., 2003; Gérard et al., 2004; Goellner et al., 2006; Ouellette et al., 2006; Mäurer et al., 2007; Brinkworth et al., 2018). Among the different models of *Chlamydia* persistence, we will focus on the two that have been most thoroughly studied: the beta-lactam antibiotic penicillin and IFNγ, a cytokine that is critical for innate and adaptive immunity.

## *Chlamydia* Persistence as a Response to Penicillins

As a member of the beta-lactams family of antibiotics, penicillins exert their antimicrobial activity by blocking the cross-linking of the peptidoglycan, a key step in cell wall synthesis in bacteria. The general mechanism of action for beta-lactam antibiotics is the inactivation of penicillin binding proteins (PBPs) by targeting their transpeptidase domain. PBPs catalyze the last step in peptidoglycan synthesis, which is the cross-linking of pentapeptide sidechains by a transpeptidation reaction. Due to the structural similarity of beta-lactams with the terminal D-Ala-D-Ala of the pentapeptide precursor, which is the natural substrate of PBPs, these antibiotics form a stable acyl-enzyme covalent bond with PBPs abolishing their peptidoglycan cross-linking activity (Sauvage et al., 2008). As these cross-links are pivotal for conferring strength and rigidity to both, Gram-positive and Gram-negative bacterial cell walls, beta-lactams usually lead to bacteria lysis due to high internal osmotic pressures.

Exposure to penicillin in cell culture provided the first evidence for *Chlamydia* persistence and the first *in-vitro* models to study this phenomenon (reviewed in Beatty et al., 1994b). Under these conditions, EB to RB transition is not prevented but once in the RB stage, cell division is blocked, inclusions are small and populated by only a few enlarged RBs (aRBs) and there is a halt in RB to EB differentiation until the antibiotic is removed (Galasso and Manire, 1961; Matsumoto and Manire, 1970; Skilton et al., 2009). Another feature of penicillin-induced persistence is that the inhibition of cell division is accompanied by accumulation of 16s rRNA and only a partial reduction in replication of genomic or plasmidic DNA (Lambden et al., 2006; Ouellette et al., 2006; Skilton et al., 2009; Kintner et al., 2014). Remarkably, a detailed video-microscopy analysis of *C. trachomatis* development during penicillin-induced persistence showed that in the recovery phase, even though aRBs remain observable inside the inclusion, normal RBs are generated from them in an asynchronous manner through a budding-resembling process (Skilton et al., 2009).

A relatively recent study carried out a careful analysis of the effect caused by beta-lactams on *C. trachomatis* serovar E (Kintner et al., 2014). In this study, HeLa cells monolayers were infected with *C. trachomatis* exposed to a variety of beta-lactam antibiotics at physiologically relevant concentrations, including 6 different penicillins (penicillin G, penicillin V, amoxicillin, ampicillin, carbenicillin, and piperacillin). Addition of any of the penicillins tested at the time of infection, did not prevent inclusion formation even at concentrations 100 times higher than the physiological serum concentration. However, inclusions were small and contained aRBs. Also, Kintner et al. demonstrated that if penicillins are added at 24 h post-infection, when inclusions are already established, the formation of aRBs is induced and production of infectious progeny is reduced by 95%. Noticeably, beta-lactams not belonging to the penicillin group, like the monobactam aztreonam and the cephalosporins ceftriaxone or cefotaxime, did not seem to affect inclusion size, morphology or infectious progeny generation, indicating that not all beta-lactams trigger the same response (Kintner et al., 2014). The relevance of these observations is that they could mimic clinically important scenarios that occur when patients asymptomatically infected with *Chlamydia*, are prescribed with beta-lactams due to other concomitant infections.

*Chlamydia* encode peptidoglycan-synthesizing high molecular weight PBPs (PBP-2 and PBP-3) and produce peptidoglycan, indicative that they possess the natural target of beta-lactam antibiotics (Barbour et al., 1982; Ouellette et al., 2012; Jacquier et al., 2014; Packiam et al., 2015). Thus, inhibition of peptidoglycan production, leading to a disruption in cell wall synthesis, may be a stressing condition that triggers *Chlamydia* persistence in presence of these antibiotics.

**Figure 2:**
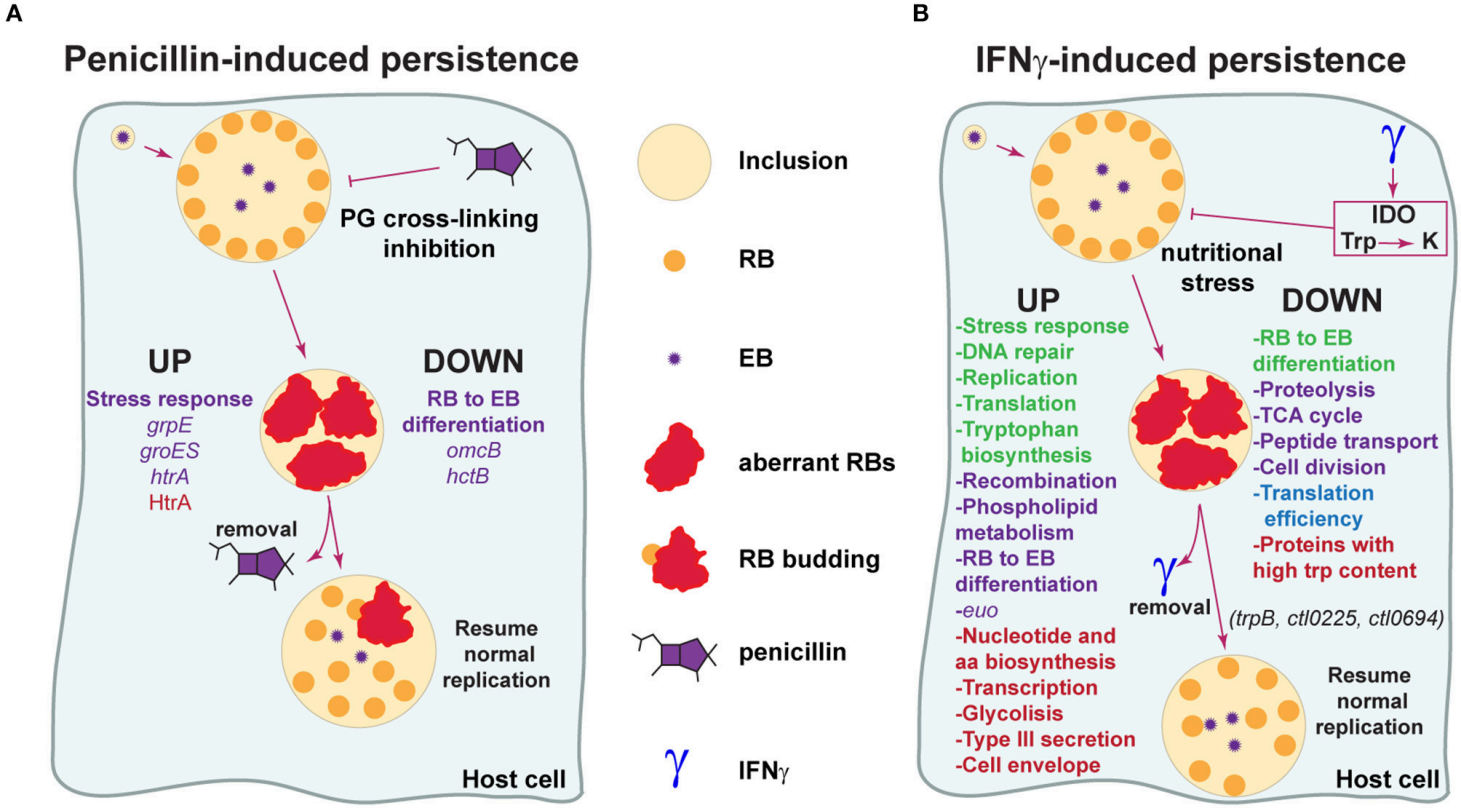
Model for the molecular basis of *Chlamydia* evasion of antimicrobial activity elicited by penicillins and IFNγ. **(A)** Penicillins inhibit crosslinking of the peptidoglycan (PG), leading to disruption of cell wall synthesis. *Chlamydia* respond to this stress by downregulating genes required for RB to EB transition (*omcB, hctB*), halting cell division and adopting an aberrant, enlarged morphology (aberrant RB) (Goellner et al., 2006; Ouellette et al., 2006). Genes associated to stress responses are upregulated (*grpE, groES, htrA*) (Goellner et al., 2006; Di Pietro et al., 2012). During this stage, the bacteria is able to “persist” for long periods of time in a viable but non-cultivable state and infectious progeny generation is abrogated. When penicillin is removed, *Chlamydia* exit persistence and resume generation of normal RBs, which may originate from aRBs through a budding-like process. The protease/chaperone protein HtrA may participate in reversion from penicillin-induced persistence (Huston et al., 2008; Ong et al., 2013). Reported processes/genes/proteins up- and down-regulated during penicillin-induced persistence are indicated. Data based on transcriptional or protein studies are highlighted in purple and red, respectively. **(B)** IFNγ is a cytokine with key roles in host defense against pathogens, including *Chlamydia*. IFNγ activates indoleamine-2,3-dioxygenase (IDO), causing the degradation of tryptophan (Trp) into kynurenine (K) and triggering nutritional stress in *Chlamydia*. In response to this stress, *Chlamydia* enters into a persistent state analogous to what is observed with penicillin. In this state, *Chlamydia* alters gene transcription and expression in order to modify key biological processes and warrant survival. Reported processes/genes/proteins up- and down-regulated during IFNγ-induced persistence are indicated. Data based on transcriptional studies (Byrne et al., 2001; Belland et al., 2003; Goellner et al., 2006; Ouellette et al., 2006) is indicated in purple. Data based on protein studies (Molestina et al., 2002; Mukhopadhyay et al., 2006; Ouellette et al., 2006; Østergaard et al., 2016) is shown in red. Data supported by both transcriptional and protein studies is indicated in green. Data based on global activity of the translation pathway is shown in blue. Note that RB to EB differentiation transcripts have been found increased and decreased in different studies, as discussed in the manuscript. *trpB* (tryptophan synthase), *ctl0225* (a predicted small neutral amino acid transporter) and *ctl0694* (a hypothetical oxidoreductase) have been found to play a role in reversion from IFNγ-induced persistence in *C. trachomatis* (Muramatsu et al., 2016).

The molecular basis of *Chlamydia* response to stress caused by exposure to penicillins is still poorly characterized (Figure 2A). According to a transcriptional analysis carried out by Dr. Byrne's group in *C. pneumoniae* during penicillin-induced persistence, the late genes *omcB* and *hctB*, involved in RB to EB differentiation, are not induced even at 48 h post-infection, strongly suggesting that in this condition the expression profile resembles that of the RB stage (Ouellette et al., 2006). Another work revealed that late gene transcription is also downregulated during penicillin-induced persistence in *C. psittaci*, together with upregulation of the stress response genes *grpE* and *groES* (Goellner et al., 2006). Additionally, the stress response protease/chaperone HtrA and its gene, which is conserved across *Chlamydia* species, have been reported to be increased during penicillin-induced persistence (Huston et al., 2008; Di Pietro et al., 2012). In line with this, treatment with the HtrA inhibitor JO146 severely impairs production of infectious progeny during recovery from penicillin-induced persistence in *C. trachomatis*, suggesting that this protease plays a role in the reversion process (Ong et al., 2013).

A relevant question is: can observations emerged from *in-vitro* models of penicillin-induced persistence be extrapolated to events occurring in infected humans? Dr. Schoborg's group reported the first animal model to study *Chlamydia* persistence (Phillips Campbell et al., 2012). By infecting the female genital tract of amoxicillin-treated BALB/c mice with *C. muridarum*, these investigators made several significant findings: (i) *C. muridarum* enters a viable non-infectious state in the genital tract; (ii) accumulation of chlamydial pre-16s rRNA is not affected; (iii) aRBs are observed in the infected tissue and (iv) shedding of infectious particles peaks upon termination of amoxicillin treatment. These findings indicate that *Chlamydia* persistence, as defined in cell culture, can occur *in-vivo* upon amoxicillin treatment. Another relevant observation is that amoxicillin-induced persistence in mice infected intravaginally with *C. muridarum*, results in increased failure of subsequent treatment with the first choice anti-chlamydial antibiotic azithromycin (Phillips-Campbell et al., 2014). These observations are consistent with previous results obtained in cell culture infection models (Wyrick and Knight, 2004). Then, it seems a reasonable clinical consideration that penicillin-induced *Chlamydia* persistence may confer increased resistance to other antibiotics, including first choice anti-*Chlamydia* drugs.

## *Chlamydia* Persistence as a Response to IFNγ

IFNγ is a pleiotropic cytokine secreted primarily by T-lymphocytes and NK cells, with key roles in regulation of immune responses and in host defense against pathogens, including *Chlamydia* (McClarty et al., 2007; Billiau and Matthys, 2009). Initially identified as an antiviral factor (Wheelock, 1965), IFNγ exerts antimicrobial effects on *Chlamydia* (reviewed in Beatty et al., 1994b). Byrne et al. (1986) found that IFNγ restricted *C. psittaci* growth in human uroepithelial T24 cells and that this effect was due to increased catabolism of the essential amino acid tryptophan within the host cell. They proposed that depletion of intracellular pools of tryptophan was achieved by IFNγ-mediated increased activity of indoleamine-2,3-dioxygenase (IDO), a catabolic enzyme that degrades tryptophan into N-formylkynurenine and kynurenine. Indeed, increased IDO activity and tryptophan depletion are responsible for IFNγ-induced growth inhibition of *C. trachomatis* serovar B in human conjunctival epithelial cells (Rapoza et al., 1991). Soon after, a seminal paper from Beatty and collaborators discovered that in presence of IFNγ, *C. trachomatis* leads to a persistent infection “*characterized by the development of noninfectious atypical chlamydial forms, from which infectious progeny could be recovered only when IFN*γ *was removed from the culture system*” (Beatty et al., 1993). A follow-up study confirmed that IDO-mediated deprivation of tryptophan was responsible for IFNγ induction of a persistent *C. trachomatis* form in cell culture (Beatty et al., 1994a). Similar observations have been made in *C. pneumoniae* (Pantoja et al., 2001). All these findings are consistent with the idea that *Chlamydia* undergo persistence to evade the antimicrobial effects caused by IFNγ-mediated starvation of an amino acid for which these bacteria are auxotrophs. Independently of tryptophan starvation, antimicrobial effects triggered by IFNγ during *Chlamydia* infection include GTPases of the Immunity Related GTPases (IRG) and Guanylate Binding Proteins (GBP), which can restrict the replication of *C. trachomatis* in mouse and human cells, respectively (Bernstein-Hanley et al., 2006; Tietzel et al., 2009).

It is of great interest to understand the molecular basis of *Chlamydia* persistence in response to IFNγ (Figure 2B). Belland et al. cataloged *C. trachomatis* serovar D transcripts that were up- and down-regulated in an IFNγ-induced persistence and reactivation model of infection in HeLa cells (Belland et al., 2003). These investigators showed that in presence of IFNγ, *Chlamydia* genes involved in tryptophan biosynthesis, DNA repair and recombination, phospholipid metabolism, stress response, and protein translation were up-regulated. On the other hand, genes related to proteolysis, peptide transport, the TCA cycle, cell division and RB to EB differentiation were down-regulated. In addition, the early gene *euo*, which has been proposed as a repressor of late genes transcription (Rosario and Tan, 2012), was found highly upregulated during IFNγ-induced persistence both in *C. trachomatis* and *C. pneumoniae* (Belland et al., 2003; Ouellette et al., 2006). Notably, the transcriptome of *C. trachomatis* during IFNγ-induced persistence is in agreement with well-established properties attributed to aRBs: a halt in cell division and infectious progeny generation. Similarly, work from Byrne et al. revealed that genes involved in chromosome replication, repair and recombination are transcribed during IFNγ-induced persistence in *C. pneumoniae*-infected HEp2 cells, while genes related to cytokinesis are downregulated (Byrne et al., 2001). One transcriptional study reported that genes involved in RB to EB differentiation were upregulated in *C. pneumoniae* in presence of IFNγ (Ouellette et al., 2006). Even though this is not in full agreement with previous reports (Belland et al., 2003; Goellner et al., 2006), the apparent inconsistencies may be due to different normalization methods (i.e., 16s rRNA vs. *gyrA* vs. DNA content) used for transcription data analysis (Schoborg, 2011).

Because transcriptional data not always correlate with actual protein expression profiles, proteomic studies can be very useful tools to investigate the molecular basis of *Chlamydia* persistence. As shown in Figure 2B, early proteomic analysis carried out in *C. pneumoniae* during IFNγ-induced persistence, found increased levels of proteins involved in stress response, nucleotide and amino acid biosynthesis, DNA replication, transcription, translation, glycolysis, type III secretion, and cell envelope (Molestina et al., 2002; Mukhopadhyay et al., 2006). More, a recent paper used a label-free proteomics approach and carried out the first comprehensive comparison of *C. trachomatis* serovar D EBs, RBs and IFNγ-induced aRBs (Østergaard et al., 2016). Remarkably, this study uncovered that the proteome of aRBs was very similar to that of the RBs, except that they expressed very high levels of tryptophan synthase (subunits TrpA and TrpB), which can transform indol into tryptophan. This finding is in agreement with previously mentioned transcriptomic data (Belland et al., 2003). Interestingly, it has been proposed that tryptophan synthase, not functional in ocular serovars, could provide a salvage pathway against tryptophan depletion for *C. trachomatis* genital serovars, given their co-existence with indol-producing microbiota in the female genital tract (Caldwell et al., 2003; Aiyar et al., 2014). This is in line with evidence showing that TrpB from *C. trachomatis* genital serovars, is capable of utilizing indole for the biosynthesis of tryptophan (Fehlner-Gardiner et al., 2002). Indeed, a *trpB* null mutant of *C. trachomatis* serovar D can be fully recovered from IFNγ-induced persistence upon addition of tryptophan but not indole (Kari et al., 2011). Another notable finding of the mentioned proteomic study, was that aRBs expressed overall lower levels of proteins with high tryptophan content, reflecting at a proteome level, the struggle to resist tryptophan restriction imposed by IFNγ (Østergaard et al., 2016). This is in agreement with the observation that the *Chlamydiaceae* have undergone evolutionary selection for proteins with lower-than-average tryptophan content (Down-Trp selection), which may operate during the persistent state triggered by tryptophan starvation (Bonner et al., 2014). Ouellette and coworkers found that during IFNγ-induced tryptophan restriction, *C. pneumoniae* accumulates Trp-codon rich transcripts, presumably as a result of ribosome stalling on Trp-codons (Ouellette et al., 2016, 2018). This could partially explain a previous report showing that in response to tryptophan restriction, *C. pneumoniae* undergoes increased transcription but inefficient translation, pointing out to an apparent uncoupling of these two pathways (Ouellette et al., 2006).

Because *Chlamydia* have been historically refractory to genetic manipulation, the role of individual genes involved in persistence is ill-defined. Only recently, tools have become available for *Chlamydia* genetic analysis, though they are still limited compared to other bacteria (reviewed in Bastidas and Valdivia, 2016). To date, there is only one report of a systematic approach to elucidate the genetic basis of *Chlamydia* persistence (Muramatsu et al., 2016). Muramatsu et al. used a collection of ~2,000 GFP-labeled, chemically-mutagenized *C. trachomatis* LGV-L2 strains, to screen for mutants exhibiting a reduced ability to reactivate from IFNγ-induced stress. The screening consisted on infecting in parallel HeLa cells pre-treated or not for 24 h with 10 ng/mL, with each individual mutant. The untreated condition was fixed at 24 h post-infection, while de IFNγ-treated were instead washed off the cytokine and left to recover for additional 24 h in culture media supplemented with indole before fixation. By comparing the ratio of the number of inclusions formed in untreated/recovery for each mutant, 6 mutants sensitive to IFNγ-induced persistence were identified and for 3 of them the causative mutation was elucidated. These were missense mutations in TrpB (subunit of tryptophan synthase), CTL0225 (a predicted small neutral amino acid transporter), and CTL0694 (a hypothetical oxidoreductase). Interestingly, all of these mutants were able to enter into a persistent state in presence of IFNγ and failed to reactivate upon addition of tryptophan or indole, yet displayed different sensitivities to IFNγ (Muramatsu et al., 2016), highlighting the relative impact of the mutations on protein function and the potential involvement of different *Chlamydia* genes in persistence.

A general principle for the biomedical relevance of cell culture experiments, is that conditions should resemble the *in-vivo* situation as much as possible. In this context, a study investigated the influence of oxygen levels on IFNγ-induced persistence (Roth et al., 2010). Roth and collaborators reasoned that in the female genital tract, *C. trachomatis* must survive under hypoxia (oxygen concentrations ≤ 5%), while most of the *in-vitro* experiments are carried out in normoxia. According to this study, switching the oxygen levels from normoxia to hypoxia in human fallopian tube cells *in-vitro* and *ex-vivo*, correlated with a reduction in the anti-chlamydial activity of IFNγ against genital but not ocular serovars of *C. trachomatis* and with decreased levels of IDO under hypoxia (Roth et al., 2010). Another study from the same group later evaluated the activity of first-line anti-chlamydial antibiotics against *C. trachomatis* L2 in HeLa cells during IFNγ-induced persistence in hypoxia vs. normoxia (Shima et al., 2013), and observed that the efficacy of subinhibitory concentrations of azithromycin against *C. trachomatis* was moderately reduced under hypoxia. Considering that the cellular concentrations of oxygen were not measured and thus distinctions between anoxic and hypoxic states within the cells cannot be made, the physiological relevance of these observations is not clear. However, these studies highlight that experimental conditions used to investigate *Chlamydia* persistence *in-vitro* should be carefully considered, as they may have profound effects on the results, their interpretation and extrapolation for the *in-vivo* situation.

## *In-vivo* Implications of *Chlamydia* Persistence

During infection of their hosts *Chlamydia* encounters stress imposed by immune responses or eventually by administration of antimicrobial agents. In light of the experimental findings discussed above, it is reasonable to speculate that *Chlamydia* can enter a persistent state *in-vivo*. However, the evidence is so far indirect. Evidence that persistence may occur *in-vivo*, comes from direct observation of aRBs in infected tissues. For instance, by means of immunogold electron microscopy, aRBs have been observed for *C. pneumoniae* in atherosclerotic tissue from human patients (Borel et al., 2008). Similarly, aRBs were detected for *C. suis* by immunohistochemistry and immunogold electron microscopy in the gut of naturally infected pigs (Pospischil et al., 2009). Also, *C. muridarum* aRBs were present in endocervical cells using a female genital tract model of infection in mice (Rank et al., 2011; Phillips Campbell et al., 2012). Moreover, Dr. Quayle group identified the presence of aRBs in endocervical cells obtained from a woman with *C. trachomatis* cervicitis (Lewis et al., 2014). While the observation of aRBs in infected tissues does not necessarily indicate a persistent infection, the widely demonstrated ability of *Chlamydia* to cause persistent, asymptomatic, chronic infections and apparent reactivations, is strongly suggestive. A clinical observation that further support the case for *Chlamydia* persistence *in-vivo* is post-gonococcal urethritis. A proportion of patients treated with penicillin for gonorrhoeae in the 60's and the 70's developed post-gonococcal urethritis caused by *C. trachomatis* (Richmond et al., 1972). It has been reported that *C. trachomatis* is recovered in 11–50% of patients with gonococcal urethritis, and 75–100% of these patients may develop post-gonococcal urethritis after treatment with an antibiotic ineffective for *Chlamydia*, like the beta-lactams penicillin or cephalosporins (Augenbraun and McCormack, 2015). While beta-lactams may succeed in clearance of *Neisseria gonorrhoeae*, they may only induce a persistent state in *Chlamydia*. Therefore, post-gonococcal urethritis could be conceived as the clinical manifestation of *Chlamydia* reactivation after completion of the beta-lactam regime in gonorrhoeae patients co-infected with *C. trachomatis*. Another indirect evidence of reactivation is the repeated observation of apparently healed immigrants from endemic areas undergoing active trachoma decades after, in absence of an identifiable re-exposure (Thygeson, 1963). Similarly, based on mice models of infection, *C. pneumoniae* can be reactivated in the lungs weeks after the initial inoculation upon treatment with cortisone, but not in immunocompetent animals, strongly suggesting that anti-*Chlamydia* immune responses trigger these bacteria to enter into a persistent state in the infected tissues (Malinverni et al., 1995; Laitinen et al., 1996). Also, the detection of *Chlamydia* antigens, DNA or RNA in clinical specimens in the absence of cultivability, is consistent with a persistent state occurring *in-vivo* (reviewed in Hogan et al., 2004). For instance, *C. trachomatis* antigens or DNA were identified in biopsy samples from culture-negative women with post-infectious tubal infertility, even after antibiotic treatment (Patton et al., 1994). In addition, *C. trachomatis* DNA and RNA have been detected weeks after cultures were already negative in a trachoma model of infection based on cynomolgus monkeys (Holland et al., 1992). However, it should be noted that detection of relatively stable molecules such as DNA or protein antigens does not necessarily indicate the presence of viable organisms and thus is not conclusive evidence of a persistent phenotype *in-vivo*. Importantly, a recent whole-genome sequence analysis from clinical samples/isolates presented compelling evidence that the same strain of *C. trachomatis* can persist for 3–5 years in the genital tract of women, regardless of regular antibiotic treatment and with none to few genomic mutations accumulated (Suchland et al., 2017). This indicates that *Chlamydia*'s strategy to persist in the host cell is not mutational, but may instead rely on the orchestration of a specific response oriented to avoid pathogen elimination.

If persistence occurs *in-vivo*, a likely scenario is that a fraction of *Chlamydia* bacteria enters into a persistent state in the host in response to unfavorable growth conditions, like those arisen from antibiotic treatment or from IFNγ produced by immune cells in response to infection. *Chlamydia* persistence may then contribute to the establishment of chronic and minimally symptomatic infections that are considered critical for long term tissue damage and pathogenesis of chlamydial diseases.

## Perspectives and Concluding Remarks

The ability of *Chlamydia* to enter a persistent state in response to antimicrobials in cell culture has been studied for decades. This persistent state invariably includes a reversible interruption in the productive cycle, but the precise mechanism required to achieve this vary with the different inducers. Experimental models of *Chlamydia* persistence in cell culture are helpful only if they can be extrapolated to *in-vivo* infections. To date the *in-vivo* occurrence of a persistent state(s) in *Chlamydia* resembling that observed in cell culture is consistent with morphological and clinical observations. For activation of a persistence program in *Chlamydia*, the pathogen should undergo a series of events including: sensing the stress condition, activation of gene expression programs that enable entry into persistence, staying persistent, and ultimately sensing favorable conditions that enable exit from persistence to resume normal replication and transition into infectious EBs. Many critical questions regarding each of these steps remain. How is damage sensed? How is persistence regulated? How does *Chlamydia* enter into and exit from persistence? What are the genetic basis for persistence in the context of different stimuli and interactions with the host? What chlamydial genes are required for persistence and how do they participate? Can we intervene in order to prevent *Chlamydia* entry or exit from persistence? What are the consequences of such interventions for *Chlamydia* pathogenesis *in-vivo*? Emerging tools for genetic manipulations in *Chlamydia* have started a new and exciting time for *Chlamydia* research, opening the possibility to address those and other relevant questions about *Chlamydia* persistence.

## Author Contributions

HS, RV, and MP wrote the paper. HS and RV contributed with funding support.

### Conflict of Interest Statement

The authors declare that the research was conducted in the absence of any commercial or financial relationships that could be construed as a potential conflict of interest.
